# High quality draft genome sequence of the heavy metal resistant bacterium *Halomonas zincidurans* type strain B6^T^

**DOI:** 10.1186/1944-3277-9-30

**Published:** 2014-12-29

**Authors:** Ying-Yi Huo, Zheng-Yang Li, Hong Cheng, Chun-Sheng Wang, Xue-Wei Xu

**Affiliations:** 1 Laboratory of Marine Ecosystem and Biogeochemistry, Second Institute of Oceanography, State Oceanic Administration, Hangzhou, P. R. China; 2 College of Life Sciences, Zhejiang University, Hangzhou, P. R. China

**Keywords:** *Halomonas*, Heavy metal resistant, The South Atlantic Ocean, Genome

## Abstract

*Halomonas zincidurans* strain B6^T^ was isolated from a deep-sea heavy metal rich sediment from the South Atlantic Mid-Ocean Ridge. The strain showed significant resistance to heavy metals, especially to zinc. Here we describe the genome sequence and annotation, as well as the features, of the organism. The genome contains 3,325 protein-coding genes (2,848 with predicted functions), 61 tRNA genes and 6 rRNA genes. *H. zincidurans* strain B6^T^ encodes 31 genes related to heavy metal resistance. And HGT may play an important role in its adaption to the heavy metal rich environment. *H. zincidurans* strain B6^T^ may have potential applications in the bioremediation of heavy metal-contaminated environments.

## Introduction

Heavy metals, either essential (e.g. Mn, Zn, Cu, Co, Ni and Mo) or toxic (e.g. Hg, Ag and Cd), are generally harmful to microbial cells even at low concentrations, as to other living organisms [1,2]. However, some microorganisms are able to resist to certain kinds and concentrations of heavy metals through several mechanisms, such as incorporating or precipitating heavy metals into complexes, oxidizing or reducing metals to less toxic valence states, and direct transporting metals out of the cell [3,4]. These heavy metal resistant microorganisms have been attracting great interests because of their potential biotechnological applications in bio-mining of expensive heavy metals and bioremediation of heavy metal-contaminated environment [2].

*Halomonas*, the largest genus of the family *Halomonadaceae*, can be found in most saline environments, including marine environments, salterns, saline lakes and soils, as well as salty foods, etc. [5,6]. *Halomonas zincidurans* strain B6^T^, a moderately halophilic bacterium, was isolated from a deep-sea sediment from the South Atlantic Mid-Ocean Ridge [5]. The strain was able to grow in medium containing high concentrations of heavy metals, especially Zn^2+^ ion, which is not detected in the reference strains and other moderately halophiles [5,7]. Therefore, the novel isolate was named as *H. zincidurans* due to its particular resistance to zinc ion [5]. Here, we present a summary classification and a set of features of *H. zincidurans* strain B6^T^, together with the description of the genomic sequencing and annotation.

## Organism information

A deep-sea sediment sample, TVG10, was collected from the South Atlantic Mid-Ocean Ridge (Table 1). There were many small hard orange red-colored lumps mixed in the sediment sample, which might be the particles containing ferric oxide and diffusing with hydrothermal plumes [8]. Not surprisingly, the concentrations of heavy metals in sample TVG10 were much higher than those in the samples collected from deep-sea seamount sediment [9], offshore sediment [10] and continental crust [11] (Additional file 1: Table S1), including Fe (98.99 mg/g), Mn (42.48 mg/g), Cu (0.839 mg/g), Ni (0.338 mg/g), Zn (0.285 mg/g), Cr (0.195 mg/g) and Co (0.064 mg/g). With consideration of the heavy metal rich environment, marine broth 2216 medium (MB, BD) containing 20 mM Mn^2+^ was used to isolate heavy metal resistant strains. Subsequently a strain named B6^T^ was obtained [5].

**Table 1:** **Classification and general features of *H. zincidurans *** **B6**^**T **^**according to the MIGS recommendations **[12]

**MIGS ID**	**Property**	**Term**	**Evidence code**^**a**^
	Current classification	Domain *Bacteria*	TAS [13]
		Phylum *Proteobacteria*	TAS [14]
		Class *Gammaproteobacteria*	TAS [15,16]
		Order *Oceanospirillales*	TAS [15,17]
		Family *Halomonadaceae*	TAS [18]–[22]
		Genus *Halomonas*	TAS [22]–[24]
		Species *Halomonas zincidurans*	TAS [5]
		Type strain B6^T^ = CGMCC 1.12450^T^ = JCM 18472^T^	
	Gram stain	Negative	TAS [5]
	Cell shape	Rod	TAS [5]
	Motility	Motile	TAS [5]
	Sporulation	Nonsporulating	TAS [5]
	Temperature range	4-37°C	TAS [5]
	Optimum temperature	35°C	TAS [5]
	pH range; Optimum	5.0-8.5; 7.0	
	Carbon source	Adonitol, L-arabinose, cellobiose, ethanol, D-fructose, D-glucose, glycerol, maltose, mannitol, D-mannose, D-ribose, D-salicin, D-sorbitol, starch, D-xylose, acetate, citrate, D-gluconate, propionate, pyruvate, succinate, L-alanine, L-arginine, glycine, L-glutamate, L-lysine, L-ornithine and L-serine	TAS [5]
MIGS-6	Habitat	Deep-sea sediment	TAS [5]
MIGS-6.3	Salinity	Moderately halophilic, 0.5-15% NaCl	TAS [5]
MIGS-22	Oxygen	Strictly aerobic	TAS [5]
MIGS-15	Biotic relationship	Free-living	NAS
MIGS-14	Pathogenicity	Not reported	
MIGS-4	Geographic location	South Atlantic Ocean	TAS [5]
MIGS-5	Sample collection time	Feb 20, 2012	NAS
MIGS-4.1	Latitude	13.60° S	TAS [5]
MIGS-4.2	Longitude	14.52° W	TAS [5]
MIGS-4.3	Depth	2950 m	TAS [5]
MIGS-4.4	Altitude	-2950 m	TAS [5]

Evidence codes - TAS: Traceable Author Statement (i.e., a direct report exists in the literature); NAS: Non-traceable Author Statement (i.e., not directly observed for the living, isolated sample, but based on a generally accepted property for the species, or anecdotal evidence). These evidence codes are from the Gene Ontology project [25].

**Figure 1:**
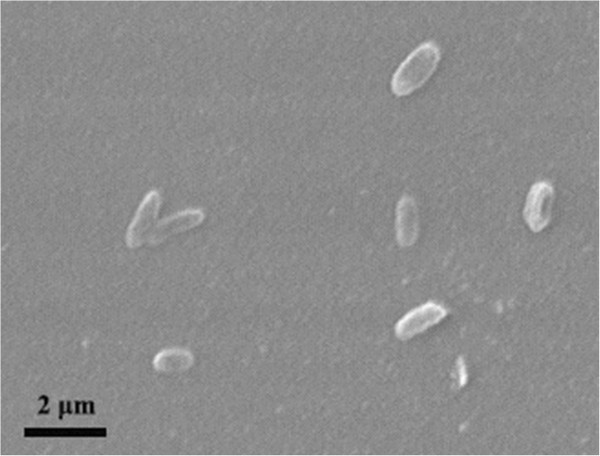
**Micrograph of *H. zincidurans *** **strain B6**^**T **^**obtained by scanning electron microscopy (S260; Cambridge).**

**Figure 2:**
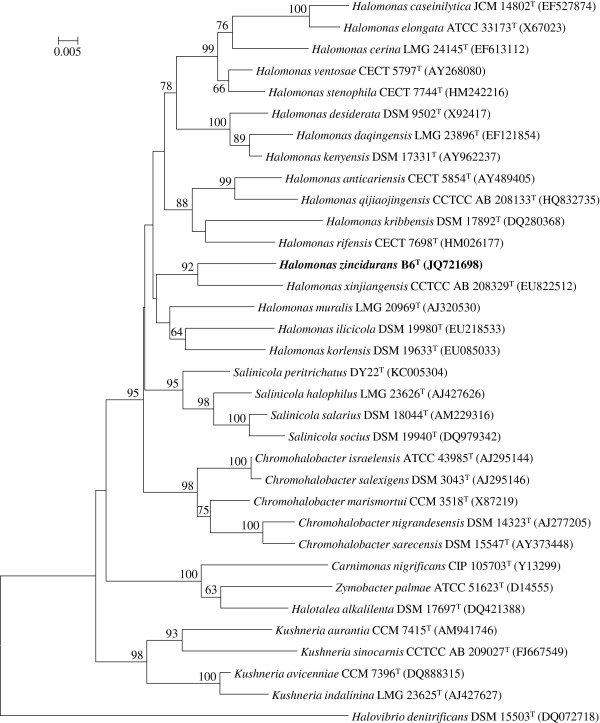
**Phylogenetic tree highlighting the position of *H. zincidurans *strain B6**^**T **^**relative to phylogenetically closely related type strains within the family Halomonadaceae.** The sequences were aligned using Clustal W [26], and the neighbor-joining tree [27] was constructed based on kimura 2-parameter distance model [28] by using MEGA5 [29]. Bootstrap values above 60% are shown obtained from 1,000 bootstrap replications. Bar, 0.05 substitutions per nucleotide position. The corresponding GenBank accession numbers are displayed in parentheses.

*H. zincidurans* strain B6^T^ is a Gram-stained negative, rod-shaped (Figure 1), moderately halophilic bacterium growing at 0.5-15% (w/v) NaCl (Table 1). Strain B6^T^ exhibited the highest 16S rRNA gene sequence similarity with *H. xinjiangensis* (96.1%). Phylogenetic analysis based on 16S rRNA gene sequences showed that strain B6^T^ and *H. xinjiangensis* clustered together in a distinct branch within the genus *Halomonas* with a high bootstrap value (Figure 2). Strain B6^T^ was able to resist high concentrations of heavy metals in liquid HM medium, including Mn^2+^ (200 mM), Co^2+^ (1.0 mM), Cu^2+^ (2.5 mM) and Zn^2+^ (14 mM). Its resistance to Zn^2+^ could be much higher (30 mM) when incubated on marine agar 2216 medium (MA, BD) [5], comparing to only 1 mM Zn^2+^ resisted by *H. xinjiangensis* TRM0175^T^. And the maximum zinc resistance concentration for 250 moderately halophilic bacteria, reported by Nieto *et al*., was only 2.5 mM [7]. Therefore, *H. zincidurans* strain B6^T^ is of significant interest due to its prominent resistance to zinc.

## Genome sequencing information

### Genome project history

The next-generation shotgun-sequencing and quality assurance was performed at the Beijing Genome Institute (BGI, Shenzhen). The gap closure and annotation processes were performed by the authors. The Whole Genome Shotgun project of *H. zincidurans* strain B6^T^ has been deposited at DDBJ/EMBL/GenBank under the accession JNCK00000000. The version described in this paper is version JNCK01000000. Table 2 presents the project information and its association with MIGS version 2.0 compliance [12].

**Table 2:** Project information

**MIGS ID**	**Property**	**Term**
MIGS-31	Finishing quality	High-quality draft
MIGS-28	Libraries used	One pair-end 494 bp library and one pair-end 2,586 bp library
MIGS-29	Sequencing platforms	Illumina HiSeq 2000
MIGS-31.2	Fold coverage	120 × (494 bp library) and 90 × (2,586 bp library)
MIGS-30	Assemblers	SOAP*denovo*[30]
MIGS-32	Gene calling method	Glimmer v3.02 [31]
	Locus Tag	HALZIN
	Genbank ID	JNCK00000000
	Genbank Date of Release	July 21, 2014
	GOLD ID	Gi0069861
	BIOPROJECT	PRJNA234075
	Project relevance	Type strain, environmental, heavy metal resistance
MIGS-13	Source Material Identifier	CGMCC 1.12450, JCM 18472

### Growth conditions and DNA isolation

*H. zincidurans* strain B6^T^ was aerobically cultivated in MB medium at 30°C. Total genomic DNA was extracted using the method described by Marmur [32]. The quality and quantity of the genomic DNA was determined by 0.6% agarose gel electrophoresis with *λ*-Hind III digest DNA marker (TaKaRa, Dalian, China) and by a Qubit® fluorometer (Invitrogen, CA, USA) with Qubit dsDNA BR Assay kit (Invitrogen, CA, USA). About 350 μg DNA with a concentration of 450 ng/μl was obtained.

### Genome sequencing and assembly

Whole-genome shotgun DNA sequencing of *H. zincidurans* strain B6^T^ was performed using Solexa paired-end sequencing technology (HiSeq2000 system, Illumina, USA) [33]. Two libraries with insert size 494 bp and 2,586 bp were constructed and a total of 519 Mb and 416 Mb raw data were produced before filtering. After removing the adapter, duplicated reads and short inserts from the data of large library, there remained 433 Mb (~120-folds genome coverage) and 328 Mb (~90-folds genome coverage) clean data from the small and large libraries for assembling, respectively. Then these sequences were assembled into 15 contigs using the SOAP*denovo* v.1.05 [30], the contig N50 length of which was 1,864,365 bp. PCR primers for gap closure were designed by Primer Premier v.5. PCR reactions were performed with PrimeSTAR HS Polymerase (TaKaRa, Dalian, China) and the amplicons were sequenced using Sanger and primer walking technologies. The sequenced fragments were subsequently assembled with the contigs using SeqMan of the Lasergene package (DNAstar, Madison, WI) into 2 contigs.

### Genome annotation

The whole genomic tRNAs were identified using tRNAscan-SE v.1.21 [34] with bacterial model, and rRNAs were found by RNAmmer v.1.2 Server [35]. ORFs were predicted using Glimmer v.3.0 [31]. The predicted ORFs were translated and analyzed using the NCBI nonredundant, Swiss-Prot [36] and COG [37] databases, as well as RAST server online [38] for genome annotation. KAAS [39] was used to assign the predict proteins into KEGG pathway [40] with BBH method. Genes with signal peptides and transmembrane helices were predicted using TMHMM server v.2.0 [41] and SignalP server v.4.1 [42], respectively. The G+C content, G+C content at the third-codon position and RSCU were calculated by CodonW v.1.4.4.

## Genome properties

**Figure 3:**
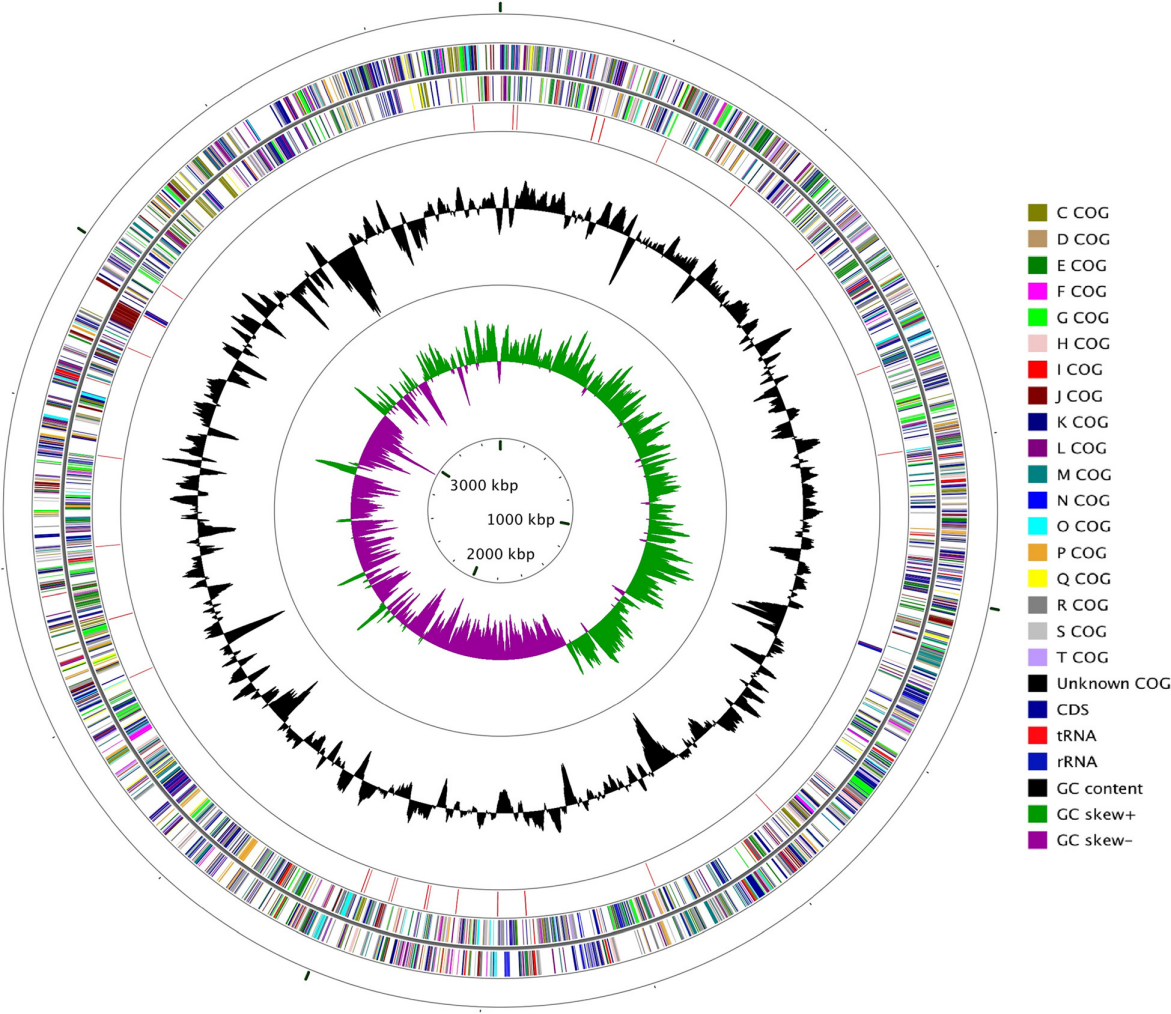
**Circular map of the chromosome of *H. zincidurans *strain B6**^**T**^**.** Labeling from the outside to the inside circle: ORFs on the forward strand (colored by COG categories), ORFs on the reverse strand (colored by COG categories), RNA genes (tRNAs red, rRNAs blue), G+C content (peaks out/inside the circle indicate values higher or lower than the average G+C content, respectively), GC skew (calculated as (G-C)/(G+C), green/purple peaks out/inside the circle indicates values higher or lower than 1, respectively).

The genome was assembled into 2 contigs, one with a size of 3,546,937 bp and the other with 7,823 bp (Table 3). The G+C content determined based on the total 3,554,760 bp sequences was 66.41%. A total of 3,392 genes were predicted, including 3,325 protein-coding genes, 61 tRNA genes and two copies of 16S-23S-5S rRNA gene operons (Table 4 and Figure 2). Among the protein coding genes, 2,848 were assigned to putative functions, and the remaining was annotated as hypothetical proteins. In total, 1,938 and 442 protein coding genes were assigned to KEGG and subsystems, respectively. The detailed properties and the statistics of the genome as well as the distribution of genes into COG functional categories are summarized in Tables 3, 4 and 5, Figure 3 and Additional file 2: Table S2.

**Table 3:** Summary of genome: two contigs

**Label**	**Size (Mb)**	**Topology**	**INSDC identifier**
Contig 1	3.546937	Linear	JNCK01000001.1
Contig 2	0.007823	Linear	JNCK01000002.1

**Table 4:** Nucleotide content and gene count levels of the genome

**Attribute**		**Genome (total)**
	**Value**	**% of total**
Genome size (bp)	3,554,760	-
DNA coding (bp)	3,153,982	88.73
DNA G+C (bp)	2,289,453	66.41
DNA scaffolds	2	-
Total genes	3,392	-
Protein coding genes	3,325	98.02
RNA genes	67	1.98
Genes with function prediction	2,916	85.97
Genes assigned to COGs	2,764	81.49
1 or more conserved domains	2,764	81.49
2 or more conserved domains	329	9.70
3 or more conserved domains	74	2.18
4 or more conserved domains	23	0.68
Genes with Pfam domains	2,188	64.50
Genes with signal peptides	180	5.31
Genes with transmembrane helices	697	20.55
CRISPR repeats	1	-

**Table 5:** Number of genes associated with the 25 general COG functional categories

**Code**	**Value**	**% of total**	**Description**
J	164	5.14	Translation
A	1	0.03	RNA processing and modification
K	230	7.21	Transcription
L	188	5.89	Replication, recombination and repair
B	4	0.13	Chromatin structure and dynamics
D	32	1.00	Cell cycle control, mitosis and meiosis
Y	-	-	Nuclear structure
V	33	1.03	Defense mechanisms
T	127	3.98	Signal transduction mechanisms
M	182	5.71	Cell wall/membrane biogenesis
N	64	2.01	Cell motility
Z	-	-	Cytoskeleton
W	-	-	Extracellular structures
U	62	1.94	Intracellular trafficking and secretion
O	109	3.42	Posttranslational modification, protein turnover, chaperones
C	215	6.74	Energy production and conversion
G	216	6.77	Carbohydrate transport and metabolism
E	325	10.19	Amino acid transport and metabolism
F	76	2.38	Nucleotide transport and metabolism
H	145	4.55	Coenzyme transport and metabolism
I	118	3.70	Lipid transport and metabolism
P	171	5.36	Inorganic ion transport and metabolism
Q	108	3.39	Secondary metabolites biosynthesis, transport and catabolism
R	391	12.26	General function prediction only
S	229	7.18	Function unknown
-	628	18.51	Not in COGs

## Insights into the genome

The genome of *H. zincidurans* strain B6^T^ contains 31 genes related to heavy metal resistance, especially to zinc resistance (Table 6). Zinc is an essential but also toxic metal for living being [2,43]. The concentration of zinc inside bacterial cells is maintained by importing limitation, efflux, accumulation and sequestration [44,45]. *H. zincidurans* strain B6^T^ possesses four heavy metal translocating P-type ATPases (HALZIN_733, HALZIN_1240, HALZIN_2196 and HALZIN_2262), which may participate in the transport of Zn^2+^, Mn^2+^, Cu^2+^, Cd^2+^, Pb^2+^, Ag + and Hg^2+^ against the concentration gradient to the periplasm [2,44]. Especially the two ZntA P-type ATPases (HALZIN_733 and HALZIN_2196) may mediate resistance to Zn^2+^, Cd^2+^ and Pb^2+^[46,47]. Zn^2+^, Co^2+^, Cu^2+^, Cd^2+^ and Ni^2+^ are able to be transported by RND family efflux transporter protein (HALZIN_54, HALZIN_1411, HALZIN_2047, HALZIN_2208 and HALZIN_2209) from both the cytoplasm and the periplasm to outside [2,44]. Usually the P-type ATPases are regulated by MerR family regulators responding to the intracellular heavy metal concentration [44,48,49]. Six analogues of MerR family regulators (HALZIN_399, HALZIN_922, HALZIN_2261, HALZIN_2264, HALZIN_2469 and HALZIN_2675) were found in the genome of *H. zincidurans* strain B6^T^. Additionally, a zinc uptake regulation protein ZUR (HALZIN_1413), which is a repressor regulator during zinc uptake, is also detected [44,50]. The presence of these genes is accordance with zinc resistance phenotype of *H. zincidurans* strain B6^T^.

**Table 6:** Description of the genes related to heavy metal resistance

**Protein id**	**Position**	**Size/aa**	**Strand**	**Predicted function**	**Closest relatives**			
					**Organism**	**Class**	**Identity**	**Accession no.**
HALZIN_54	48442-49500	352	+	RND family efflux transporter, MFP subunit	*Idiomarina sediminum*	*Gammaproteobacteria*	44%	WP_026860724
HALZIN_399	433553-434005	150	+	MerR family Cd(II)/Pb(II)-responsive transcriptional regulator	*Halomonas lutea*	*Gammaproteobacteria*	75%	WP_019019418
HALZIN_733	778272-780812	846	+	Heavy metal translocating P-type ATPase ZntA	*Gracilimonas tropica*	*Sphingobacteriia*	59%	WP_020403952
HALZIN_916	977118-976882	78	-	Mercuric transport protein MerE	*Burkholderia cepacia*	*Betaproteobacteria*	99%	YP_006965885
HALZIN_917	977480-977115	121	-	Transcriptional regulator MerD	*Pseudomonas putida*	*Gammaproteobacteria*	98%	WP_012806008
HALZIN_918	978239-977592	215	-	Alkylmercury lyase MerB	*Paraglaciecola polaris*	*Gammaproteobacteria*	84%	WP_007106069
HALZIN_919	979028-978390	212	-	Alkylmercury lyase MerB	*Paraglaciecola polaris*	*Gammaproteobacteria*	94%	WP_007106069
HALZIN_920	979808-979179	209	-	Alkylmercury lyase MerB	*Paraglaciecola polaris*	*Gammaproteobacteria*	90%	WP_007106069
HALZIN_922	980118-980540	140	+	Transcriptional regulator MerR	*Stenotrophomonas maltophilia*	*Gammaproteobacteria*	99%	WP_005413398
HALZIN_934	994405-993521	294	-	Magnesium and cobalt efflux protein CorC	*Chromohalobacter salexigens*	*Gammaproteobacteria*	81%	WP_011507633
HALZIN_1240	1334217-1331998	739	-	Heavy metal translocating P-type ATPase	*Halomonas* sp.	*Gammaproteobacteria*	97%	WP_023004666
HALZIN_1392	1499237-1498659	192	-	Superoxide dismutase	*Halomonas smyrnensis*	*Gammaproteobacteria*	85%	WP_016854901
HALZIN_1411	1521826-1522995	389	+	RND family efflux transporter, MFP subunit	*Halomonas lutea*	*Gammaproteobacteria*	76%	WP_019017686
HALZIN_1413	1526330-1526785	151	+	Zinc uptake regulation protein ZUR	*Halomonas lutea*	*Gammaproteobacteria*	82%	WP_019017691
HALZIN_2047	2179598-2182789	1063	+	RND family efflux transporter protein	*Pseudoxanthomonas suwonensis*	*Gammaproteobacteria*	85%	WP_013535339
HALZIN_2196	2338252-2335574	892	-	Heavy metal translocating P-type ATPase ZntA	*Halomonas lutea*	*Gammaproteobacteria*	65%	WP_019020337
HALZIN_2208	2355137-2351976	1053	-	RND family efflux transporter protein	*Pseudomonas alcaligenes*	*Gammaproteobacteria*	58%	WP_021217164
HALZIN_2209	2356423-2351976	428	-	RND family efflux transporter, MFP subunit	*Halomonas lutea*	*Gammaproteobacteria*	53%	WP_019020155
HALZIN_2260	2411989-2410787	400	-	Multicopper oxidase	*Sphingopyxis baekryungensis*	*Alphaproteobacteria*	55%	WP_022673021
HALZIN_2261	2412630-2413034	134	+	Transcriptional regulator MerR	*Halomonas lutea*	*Gammaproteobacteria*	90%	WP_019017365
HALZIN_2262	2413107-2415596	829	+	Heavy metal translocating P-type ATPase	*Halomonas lutea*	*Gammaproteobacteria*	92%	WP_019017357
HALZIN_2264	2416527-2416976	149	+	Transcriptional regulator MerR	*Halomonas lutea*	*Gammaproteobacteria*	89%	WP_026300314
HALZIN_2268	2423176-2423622	148	+	CopG family transcriptional regulator	*Halomonas lutea*	*Gammaproteobacteria*	80%	WP_019017364
HALZIN_2271	2424931-2425086	51	+	Copper resistance protein CopC	*Hyphomonas neptunium*	*Alphaproteobacteria*	51%	WP_011646711
HALZIN_2272	2425115-2425978	287	+	Copper resistance protein CopD	*Thialkalivibrio* sp.	*Gammaproteobacteria*	43%	WP_018881395
HALZIN_2469	2658088-2657690	132	-	Transcriptional regulator MerR	*Halomonas lutea*	*Gammaproteobacteria*	90%	WP_019020805
HALZIN_2470	2658244-2658588	114	+	Mercuric transport protein MerT	*Halomonas lutea*	*Gammaproteobacteria*	78%	WP_019020806
HALZIN_2471	2658620-2658925	101	+	Periplasmic mercury(+2) binding protein MerP	*Halomonas lutea*	*Gammaproteobacteria*	82%	WP_019020807
HALZIN_2472	2658988-2660622	544	+	Mercuric reductase, MerA family	*Halomonas lutea*	*Gammaproteobacteria*	93%	WP_019020808
HALZIN_2675	2872087-2872584	165	+	Transcriptional regulator MerR	*Halomonas* sp.	*Gammaproteobacteria*	66%	WP_023005510
HALZIN_3265	3489632-3489021	203	-	Superoxide dismutase	*Halomonas lutea*	*Gammaproteobacteria*	74%	WP_019019731

Among the 31 ORFs related to heavy metal resistance, it is noteworthy of two *mer*-operons. One *mer*-operon encodes a mercuric transport protein (MerE, HALZIN_916) for organic mercury uptake [51], a transcriptional regulator (MerD, HALZIN_917), three alkylmercury lyases (MerB, HALZIN_918-920) catalyzing organomercurials yielding Hg^2+^[52] and a transcriptional regulator (MerR, HALZIN_922). The other one encodes a transcriptional regulator (MerR, HALZIN_2469), two mercuric transport proteins (MerT and MerP, HALZIN_2470-2471) for inorganic mercury uptake [51] and a mercuric reductase (MerA, HALZIN_2472) catalyzing Hg^2+^ to Hg^0^[53]. According to the genomic data, *H. zincidurans* strain B6^T^ is able to survive in both inorganic and organic mercury environments. Interestingly, the four ORFs of the inorganic *mer*-operon showed the highest sequence identities to those of *Halomonas lutea*. Nevertheless, all the six ORFs of the organic *mer*-operon did not show the highest sequence identities to those of the genus *Halomonas*, but to the genera *Burkholderia*, *Pseudomonas*, *Gladiecola* and *Stenotrophomonas*, which indicates that the organic *mer*-operon might be acquired by HGT. Of special interest are the three alkylmercury lyases (MerB, HALZIN_918-920), which had obvious differences between the G+C content (56.6%; 57.1, 56.6 and 56.0% for these three gene sequences, respectively) as well as the G+C content at the third-codon positions (60.3%; 60.4, 61.0 and 59.4% for these three gene sequences, respectively) and those of the total protein-coding genes (65.4 and 82.8%, respectively). Besides, the RSCUs of nearly half of the 59 codons used by the three genes (23, 27 and 26 codons for HALZIN_918-920, respectively) change more than 2 folds, compared with those used by total protein-coding genes. 13 of the 31 ORFs (41.9%) were not related to *Halomonadaceae* genes according to the gene sequence similarity analysis, 9 of the 13 ORFs had RSCU change larger than 2 folds in more than 25% codons. These results indicated the existence of HGT events among the heavy metal resistance-related genes. Thus, HGT events might be an important way for *H. zincidurans* strain B6^T^ to acquire heavy metal resistant ability and to adapt to the heavy metal rich environment.

## Conclusion

The draft genome sequence of the heavy metal resistant bacteria *H. zincidurans* strain B6^T^ isolated from the South Atlantic Mid-Ocean Ridge provide an insight into the genomic basis of its heavy metal resistance ability. And HGT may play an important role in its adaption to the heavy metal rich environment. On the basis of analysis and characterization of genome, *H. zincidurans* strain B6^T^ might be resistant more kinds of heavy metal than we tested, such as Hg^2+^, Cd^2+^, Pb^2+^, Ni^2+^ and Ag^+^, etc. And it may have the potential for the bioremediation of multi-metal-contaminated environments. In addition, further analysis will be performed to confirm its resistant ability to other heavy metals and determine the mechanism of heavy metal resistance that we don’t know yet.

## Abbreviations

HGT: Horizontal gene transfer; RSCU: Relative synonymous codon usage.

## Competing interests

The authors declare that they have no competing interests.

## Authors’ contributions

YH designed and performed experiments, analyzed data and wrote the paper; ZL performed experiments; HC analyzed genome data; CW analyzed data; XX designed the experiments and wrote the paper. All authors read and approved the final manuscript.

## Supplementary Material

Additional file 1: Table S1 Concentrations of heavy metals in deep-sea sediment collected from the South Atlantic Mid-Ocean Ridge (1) and the sediments from the Central Pacific seamount (2), offshore sediment (3) and continental crust (4). Click here for file

Additional file 2: Table S2 Associated MIGS record. Click here for file
