# Supplementary material for: High quality draft genome sequence of the heavy metal resistant bacterium Halomonas zincidurans type strain B6T
Source: Stand Genomic Sci. 2014 Dec 29;9:30. doi: 10.1186/1944-3277-9-30 (PMC4286145; doi:10.1186/1944-3277-9-30)
Supplement: Additional file 1: Table S1 — Concentrations of heavy metals in deep-sea sediment collected from the South Atlantic Mid-Ocean Ridge (1) and the sediments from the Central Pacific seamount (2), offshore sediment (3) and continental crust (4). [file 1944-3277-9-30-S1.doc]

|  | 1 | 2 | 3 | 4 |
| --- | --- | --- | --- | --- |
| Fe /mg g-1 | 98.99 | 28.98 | 32.13 | 43.96 |
| Mn /mg g-1 | 42.48 | 2.60 | 0.95 | 0.79 |
| Cu /μg g-1 | 839 | 134 | 20 | 25 |
| Ni /μg g-1 | 338 | 74 | 27 | 56 |
| Zn /μg g-1 | 285 | 83 | 72 | 65 |
| Cr /μg g-1 | 195 | 57 | 43 | 126 |
| Co /μg g-1 | 64 | 52 | 14 | 24 |
