# Supplementary material for: High quality draft genome sequence of the heavy metal resistant bacterium Halomonas zincidurans type strain B6T
Source: Stand Genomic Sci. 2014 Dec 29;9:30. doi: 10.1186/1944-3277-9-30 (PMC4286145; doi:10.1186/1944-3277-9-30)
Supplement: Additional file 2: Table S2 — Associated MIGS record. [file 1944-3277-9-30-S2.doc]

| **MIGS-ID** | field name | description |
| --- | --- | --- |
| **MIGS-1** | Submit to INSDC/Trace archives |  |
| **1.1** | PID | JNCK00000000, PRJNA234075, Gi0069861 |
| **1.2** | Trace Archive | NCBI/WGS |
| **MIGS-2** | MIGS CHECK LIST TYPE |  |
| **MIGS-3** | Project Name | Genome sequencing of *Halomonas zincidurans* B6 |
| **MIGS-4** | Geographic Location | South Atlantic Mid-Ocean Ridge |
| **4.1** | Latitude | 13.60 °S |
| **4.2** | Longitude | 14.52 °W |
| **4.3** | Depth | 2,950 m |
| **4.4** | Altitude | -2,950 m |
| **MIGS-5** | Time of Sample collection | Feb. 20, 2012 |
| **MIGS-6** | Habitat (EnvO) |  |
| **6.1** | Temperature | Not reported |
| **6.2** | pH | Not reported |
| **6.3** | Salinity | Not reported |
| **6.4** | Chlorophyll | Not reported |
| **6.5** | Conductivity | Not reported |
| **6.6** | light intensity | Not reported |
| **6.7** | dissolved organic carbon (DOC) | Not reported |
| **6.8** | Current | Not reported |
| **6.9** | atmospheric data | Not reported |
| **6.10** | Density | Not reported |
| **6.11** | Alkalinity | Not reported |
| **6.12** | dissolved oxygen | Not reported |
| **6.13** | particulate organic carbon (POC) | Not reported |
| **6.14** | Phosphate | Not reported |
| **6.15** | Nitrate | Not reported |
| **6.16** | Sulfates | Not reported |
| **6.17** | Sulfides | Not reported |
| **6.18** | primary production | Not reported |
| **MIGS-7** | Subspecific genetic lineage | Not reported |
| **MIGS-9** | Number of replicons | 1 |
| **MIGS-10** | Extrachromosomal elements | Not reported |
| **MIGS-11** | Estimated Size | 3.55 Mb |
| **MIGS-12** | Reference for biomaterial or Genome report | Not reported |
| **MIGS-13** | Source material identifiers | CGMCC 1.12450 and  JCM 18472 |
| **MIGS-14** | Known Pathogenicity | Non-pathogenic |
| **MIGS-15** | Biotic Relationship | Free-living |
| **MIGS-16** | Specific Host | Not reported |
| **MIGS-17** | Host specificity or range (taxid) | Not reported |
| **MIGS-18** | Health status of Host | Not reported |
| **MIGS-19** | Trophic Level | Decomposer |
| **MIGS-22** | Relationship to Oxygen | Strictly aerobic |
| **MIGS-23** | Isolation and Growth conditions | Marine agar 2216 (MA, BD) at 28 ºC |
| **MIGS-27** | Nucleic acid preparation | SDS-Proteinase K, phenol and chloroform |
| **MIGS-28** | Library construction |  |
| **28.1** | Library size | 494 bp and 2,586 bp |
| **28.2** | Number of reads | 48,111,112 (494 bp library) and 3,648,984 (2,586 bp library) |
| **28.3** | Vector | No vector |
| **MIGS-29** | Sequencing method | Illumina HiSeq 2000 |
| **MIGS-30** | Assembly |  |
| **30.1** | Assembly method | SOAP*denovo*, K = 51 |
| **30.2** | estimated error rate | 0.002 |
| **30.3** | method of calculation | *De Brujin* graph |
| **MIGS-31** | Finishing strategy |  |
| **31.1** | Status | High-quality draft |
| **31.2** | Coverage | 120 × (494 bp library) and 90 × (2,586 bp library) |
| **31.3** | Contigs | 2 |
| **MIGS-32** | Relevant SOPs | Not reported |
| **MIGS-33** | Relevant e-resources | PMID: 23811134; GenBank No.: JNCK00000000 |
